# Risk Factors of Patient-Related Safety Events during Active Mobilization for Intubated Patients in Intensive Care Units—A Multi-Center Retrospective Observational Study

**DOI:** 10.3390/jcm10122607

**Published:** 2021-06-13

**Authors:** Hajime Katsukawa, Kohei Ota, Keibun Liu, Yasunari Morita, Shinichi Watanabe, Kazuhiro Sato, Kenzo Ishii, Daisetsu Yasumura, Yo Takahashi, Takafumi Tani, Hitoshi Oosaki, Tomoya Nanba, Ryo Kozu, Toru Kotani

**Affiliations:** 1 Department of Scientific Research, Japanese Society for Early Mobilization, 1-2-12-2F Kudan-kita, Chiyoda-ku, Tokyo 102-0073, Japan; 2 Department of Emergency and Critical Care Medicine, Graduate School of Biomedical and Health Sciences, Hiroshima University, 1-2-3 Kasumi, Minami-ku, Hiroshima 734-8551, Japan; kota@hiroshima-u.ac.jp; 3 Critical Care Research Group, The Prince Charles Hospital, 627 Rode Rd, Chermside, Brisbane, QLD 4032, Australia; keiliu0406@gmail.com; 4 Department of Emergency and Intensive Care Medicine, National Hospital Organization Nagoya Medical Center, 4-1-1 Sannomaru, Naka-ku, Nagoya, Aichi 460-0001, Japan; moltlyme2@yahoo.co.jp; 5 National Hospital Organization Nagoya Medical Center, Department of Rehabilitation Medicine, 4-1-1 Sannomaru, Naka-ku, Nagoya, Aichi 460-0001, Japan; billabonghonor@yahoo.co.jp; 6 Department of Pulmonology, Japanese Red Cross Nagaoka Hospital, Senshu-2 297-1, Nagaoka, Niigata 940-2085, Japan; satkazu@nagaoka.jrc.or.jp; 7 Intensive Care Unit, Department of Anesthesiology, Fukuyama City Hospital, 3-8-5 Zao-cho, Fukuyama, Hiroshima 721-8511, Japan; keishii1101@gmail.com; 8 Department of Rehabilitation, Naha City Hospital, 2-31-1 Furujima, Naha, Okinawa 902-8511, Japan; yasumuradai@yahoo.ne.jp; 9 Department of Healthcare Administration and Management, Graduate School of Medical Sciences, Kyushu University, 3-1-1, Maidashi, Higashi-ku, Fukuoka 812-8582, Japan; 10 Yuuai Medical Center, Department of Rehabilitation, 50-5 Yone, Tomigusuku, Okinawa 901-0224, Japan; yo.takahashi7448@gmail.com; 11 Department of Rehabilitation, Japanese Red Cross Ishinomaki Hospital, 71 Nishimichishita, Hebita, Ishinomaki, Miyagi 986-8522, Japan; takafumitani0224@gmail.com; 12 Department of Rehabilitation, Japanese Red Cross Maebashi Hospital, 389-1 Asakura-cho, Maebashi Gunma 371-0811, Japan; winegood21@yahoo.co.jp; 13 Department of Rehabilitation, Yao Tokushukai General Hospital, 1-17 Wakakusa-cho, Yao, Osaka 581-0011, Japan; nanbatomoya@yahoo.co.jp; 14 Department of Rehabilitation Medicine, Nagasaki University Hospital, 1-7-1 Sakamoto, Nagasaki 852-8520, Japan; ryokozu@nagasaki-u.ac.jp; 15 Department of Physical Therapy Science, Nagasaki University Graduate School of Biomedical Sciences, 1-7-1 Sakamoto, Nagasaki 852-8501, Japan; 16 Department of Intensive Care Medicine, Showa University School of Medicine, 1-5-8 Hatanodai, Shinagawa-ku, Tokyo 142-8666, Japan; trkotani@med.showa-u.ac.jp

**Keywords:** early mobilization, rehabilitation, safety, adverse event, mechanical ventilation, tracheally intubated patient, barrier, risk factor, post-intensive care syndrome, critical illness

## Abstract

The aim of this study is to clarify the incidence and risk factors of patient-related safety events (PSE) in situations limited to intubated patients in which active mobilization, such as sitting on the edge of the bed/standing/walking, was carried out. A multi-center retrospective observational study was conducted at nine hospitals between January 2017 and March 2018. The safety profiles and PSE of 87 patients were analyzed. PSE occurred in 10 out of 87 patients (11.5%) and 13 out of 198 sessions (6.6%). The types of PSE that occurred were hypotension (8, 62%), heart rate instability (3, 23%), and desaturation (2, 15%). Circulation-related events occurred in 85% of overall cases. No accidents, such as line/tube removal or falls, were observed. The highest incidence of PSE was observed during the mobilization level of standing (8 out of 39 sessions, 20.5%). The occurrence of PSE correlated with the highest activity level under logistic regression analysis. Close vigilance is required for intubated patients during active mobilization in the standing position with regard to circulatory dynamics.

## 1. Introduction

Critically ill patients, especially mechanically-ventilated patients, experience impaired physical functioning and quality of life following their discharge from the Intensive Care Unit [1]. As a measure to counteract this, mobilization is regarded as being beneficial for improving physical functioning as well as shortening the length of mechanical ventilation and the hospital length of stay [2,3,4,5,6,7,8]. The issue of mobilization safety has been examined in numerous studies, which have found an occurrence rate for patient-related safety events (PSE) of 2.6%, and virtually no occurrence of severe, life-threatening events [9]. However, medical instability comprising PSE has been reported as a barrier at the time of mobilization [10,11], and even when the event is not life-threatening, its occurrence is inexpedient for the patient. In fact, Sakai et al. report that in situations where a patient is forced to step down from a planned goal, in 54.6% of cases the reason for having to step down is medical instability [12]. Accordingly, identifying situations in which PSE occurs and clarifying the risk factors for PSE occurrence provides beneficial information for avoiding PSE occurrence.

Several previous studies reported situations in which PSE has occurred. However, these studies also included non-mechanically-ventilated patients and in-bed exercise, [13,14,15,16,17,18,19,20,21,22] and did not report on the occurrence of PSE in situations limited to intubated patients or active mobilization, such as sitting on the edge of the bed/standing/walking. It is possible that these studies underestimated the risk of PSE occurrence during active mobilization of intubated patients. In addition, carrying out active mobilization with tracheally intubated patients, who present the highest risk, may in fact provide the most potential benefit to such patients. For this reason, this study focused on PSE occurrence among intubated patients undergoing active mobilization, with the aim of clarifying the incidence and risk factors of PSE using data from multiple hospitals.

## 2. Materials and Methods

### 2.1. Study Design

This research is a multi-center retrospective observational study conducted at nine hospitals in Japan. Each participating hospital obtained ethical approval for the study from the relevant review board (Japanese Red Cross Nagaoka Hospital Institutional Review Board; approval code: 2130). The study was conducted in accordance with the Declaration of Helsinki. Hospital background information is provided in a supplemental table (Table S1). Protocols for sedation, analgesia, and mechanical ventilator weaning were not shared, but a Mobilization Protocol (Table S2), formulated using the protocol of Morris et al. for reference [2], was shared among all the participating hospitals. To ensure the establishment of this protocol, a six-month preparation period was provided for staff training, and exercises were standardized. At each hospital, mobilization was carried out by either a physician, nurse, physical therapist, or occupational therapist, with mobilization carried out for 20 min or more in each session. For this study, “active mobilization” was defined under the Mobilization Protocol as “sitting on the edge of the bed/standing/walking of Level 3 or higher”.

### 2.2. Patient Population

**Figure 1:**
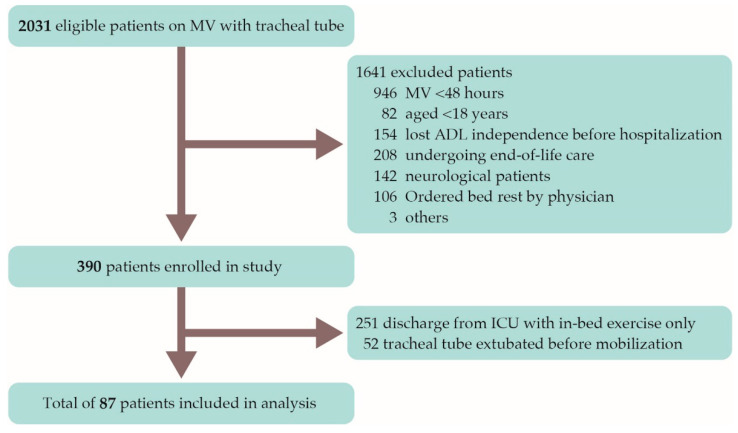
Flowchart of patient recruitment. MV: mechanical ventilation; ADL: activities of daily living; ICU: Intensive Care Unit.

The subjects of this study were 2031 tracheally intubated patients on mechanical ventilation who were admitted to the ICU at a participating hospital between January 2017 and March 2018. Using previous study for reference, inclusion and exclusion criteria were carefully defined [2]. The study excluded patients who were mechanically ventilated for less than 48 hours in the ICU; patients aged under 18 years; patients who had lost ADL independence before hospitalization; patients receiving end-of-life care; neurological patients; and patients ordered bed rest by their physician. Of the 390 patients enrolled in the study, patients who were discharged from the ICU with in-bed exercise only and patients who were extubated prior to active mobilization were withdrawn from the study, and the remaining 87 patients were analyzed (Figure 1).

### 2.3. Study Outcomes

The following data were collected from the medical records of each patient. Hospital and ICU length of stay, duration of mechanical ventilation, time to first rehabilitation session, and time to first out of bed were calculated. Number of sessions, highest activity level of mobilization, PSE details and occurrence rate, and timing of PSE occurrence were investigated as items related to mobilization safety. The highest activity of mobilization was recorded using a 5-level scale based on our protocol (Table S2) [23]. Sessions that were suspended because of PSE occurring during active mobilization were recorded. Data were also collected at the time mobilization was started in each session on the patient’s vital signs, the parameters of mechanical ventilation, the number of patients on vasopressors before active mobilization and continuous renal replacement therapy, the Richmond Agitation Sedation Scale (RASS), and the professional carrying out the mobilization.

### 2.4. Definition of Patient-Related Safety Events

Based on the criteria of previous studies that examined the safety of early mobility, ten types of patient-related safety events were defined: death, cardiopulmonary arrest, fall, removal of line, abnormal respiratory rate (<5 breaths per minute >40 breaths per minute), desaturation (<90% or 10% or more drop below baseline), heart rate instability (<40 beats per minute >130 beats per minute), systolic blood pressure instability (<80 mmHg or >200 mmHg), newly occurring arrhythmia, and other [14,16,24]. Based on the findings of these previous studies, PSE was diagnosed when medical instability continued for one minute or longer.

### 2.5. Statistical Analysis

Descriptive statistics were performed as counts and percentages, with median and interquartile range (IQR) as appropriate. Patients were divided into two groups: those who experienced PSE during a mobilization session and those who did not experience PSE. With regard to PSE occurrence rates, the occurrence rate per 1000 mobilization sessions was calculated by type of patient-related safety event and by timing of PSE occurrence. The PSE occurrence rate by event was calculated by dividing the number of PSE occurrences by all mobilization sessions, and the PSE occurrence rate by timing of PSE occurrence was calculated by dividing the number of PSE occurrences at each activity level by the number of total sessions at the same activity level. Basic patient information, mobilization-related information, and PSE occurrence details for the two groups were compared. The Fisher’s exact test and chi-square test were used for nominal variables, and the Mann–Whitney U test was used for continuous and ordinal variables. Statistical significance was defined as a two-sided *p* < 0.05. Logistic regression analysis was performed to extract risk factors associated with PSE. Under univariate analysis, variables with a significance probability of less than 5% were entered into a multivariate analysis model and the odds ratio was calculated. Statistical analysis was carried out using EZR (Version 1.37, Saitama Medical Center, Jichi Medical University, Saitama, Japan) [25]. Figures were illustrated using Illustrator 2021 (Version 25.2.3, Adobe, San Jose, CA, USA).

## 3. Results

There were 87 patients with whom it was possible to carry out active mobilization while the patient was tracheally intubated (Figure 1), and PSE occurred with 10 (11.5%) of these patients. There were no differences in baseline characteristics between the group of patients with PSE and the group of patients without PSE (Table 1). Among the group of patients with PSE, the time to first mobility session (Group without PSE: 2.0 days (1.0–4.0) versus Group with PSE: 1.0 day (0.0–1.0) Median (IQR); *p* < 0.01) and the time to first getting out of bed (4.0 days (3.0–7.0) versus 2.5 days (2.0–4.5); *p* < 0.05) were significantly shorter. The occurrence rate of PSE among cases in which first mobilization was carried out on the day, or first day of ICU admission, was significantly higher than for cases in which first mobilization was carried out on the second day or a subsequent day (25% versus 3.6%; *p* < 0.05).

Looking at the analysis results based on sessions, of the 198 active mobilization sessions carried out while the patient was tracheally intubated, PSE occurred in 13 sessions (6.6%) (Table 2). Results of the comparison between the Group with PSE and the Group without PSE showed that the highest activity level was significantly higher for the Group with PSE (Group without PSE: 3 (3–3) versus Group with PSE: 4 (4–4); *p* < 0.001), and the RASS value was also higher (−1(−1–0) versus 0(0–0); *p* < 0.05). Of the patients in the Group with PSE, 46% were administered vasopressors before mobilization, which was a significantly higher percentage than that of the Group without PSE (18%) (*p* < 0.05). In addition, for the Group with PSE the rate of participation of a physical therapist in mobilization sessions was significantly lower than for the Group without PSE (95% versus 77%; *p* < 0.05).

**Figure 2:**
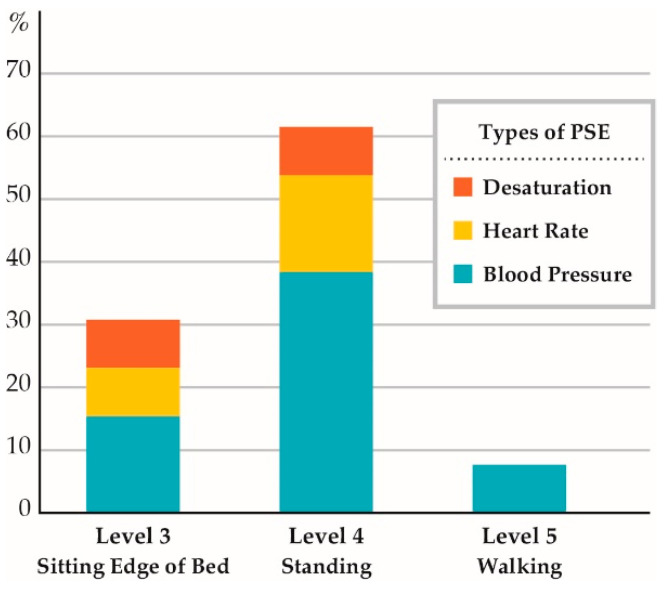
Occurrence rate of patient-related safety events (PSE) classified by mobility level.

Regarding the types of PSE that occurred, systolic blood pressure instability occurred in 8 events (62%, Event rate = 40.4 per 1000 sessions), heart rate instability occurred in 3 events (23%, Event rate = 15.2 per 1000 sessions), desaturation occurred in 2 events (15%, Event rate = 10.1 per 1000 sessions), and 2 patients experienced multiple PSE (Table 3). There were no accidents, such as line/tube removal or falls, or any severe, life-threatening events. Calculating the PSE occurrence rate by mobilization level, the highest occurrence rate was for standing (Event rate = 205.1 per 1000 sessions), followed by sitting on the edge of the bed (Event rate = 26.1 per 1000 sessions) and walking (Event rate = 166.7 per 1000 sessions). PSE while standing comprised 61.5% of all the PSE that occurred (Figure 2). The results of logistic analysis indicated a correlation between the highest activity level and PSE occurrence (Table 4).

## 4. Discussion

This research is a multi-center study to examine the incidence and risk factors of PSE, focusing on active mobilization of intubated patients in the ICU. The PSE evaluated in this research are consistent with those evaluated in previous studies [14,16,24]. The PSE occurrence rate was 11.5% by patient and 6.6% by session. In all PSE events, medical instability was non-life-threatening. These occurrence rates were higher than in a previous review conducted by Nydahl et al. that included non-mechanically ventilated patients and patients with in-bed exercise [9].

Analyzing PSE occurrence from the perspective of the day on which mobilization was started, among the Group with PSE the time to first mobility sessions and time to first getting out of a bed were shorter. Furthermore, the frequency of PSE occurrence was significantly higher among patients mobilized on the day or first day of ICU admission. Because this occurrence of PSE poses a barrier to mobilization, thorough consideration needs to be given to its prevention [26]. When mobilization is started on the day or first day of ICU admission, extra vigilance may be necessary due to the increased risk of PSE.

Regarding the results of the comparison between the Group with PSE and the Group without PSE, differences between the groups were found for the values of highest activity level, RASS, patients on vasopressors before active mobilization, and participation of a physical therapist in the mobilization. The RASS score values for the Group with PSE were close to zero. Although it had been anticipated that circulation would be stable when consciousness-level awakening was obtained, circulation-related PSE occurred in 85% of cases overall. Looking more closely at the patients with PSE, vasopressors had been administered before mobilization to 46% of these patients. Rebel et al. reported that mobilization of patients administered with vasopressors poses a higher risk [27]. Furthermore, Pohlman et al. suggested administration of vasopressors as a potential barrier to mobilization [16]. When mobilizing patients who have been administered vasopressors, careful evaluation may be necessary in setting mobilization levels.

Regarding the results of logistic regression analysis, the highest activity level was found to correlate with PSE occurrence. In addition, the results of analysis by mobilization level showed that PSE occurred most often when the patient was standing. These results indicate that high levels of mobilization, especially standing, may cause PSE to occur most frequently. Several previous studies have reported PSE occurring most frequently when the patient was in a standing position, supporting the results of this study. Lee et al. reported that PSE while standing comprised 57.7% of all PSE that they observed—the highest occurrence rate for any of the activities [24]. Sarfati et al. reported that the occurrence rate for adverse events while patients were in a sitting position was 8%, but increased to 13% among the same patient group when the patients were in a standing position. These findings support the validity of our study’s results [28].

As countermeasures against circulation-related PSE, ingestion of water [29,30], fluid infusion [31], elastic bandages [32,33], and standing up slowly [34,35] should be considered in combination. Being vigilant about the occurrence of PSE when patients are standing and implementing countermeasures can be expected to contribute to the prevention of PSE occurrence and overcoming barriers to mobilization. The findings of this study may also be helpful in improving the low rate of implementation for mobilization that has been reported in various studies [36,37,38].

This study has several limitations. Firstly, the research was a retrospective study observing a small number of cases, meaning that the results may not generalize to other settings. Secondly, the early mobilization protocol was common to all participating hospitals, but sedation/analgesia/mechanical ventilator weaning protocols were not shared. Thirdly, it is possible that selection bias may exist because the physician’s order for bed rest (e.g., cases in which the physician decides the patient’s severity themselves) was included in the criteria for excluding a patient from the study. Fourthly, the results were obtained at hospitals where medical staff were well trained, and it is therefore possible that even more PSE will occur when mobilization activities are introduced at facilities in which there are no staff with mobilization experience. In order to overcome these limitations, multi-center prospective studies examining more cases need to be carried out moving forward.

## 5. Conclusions

The PSE occurrence rate during active mobilization among tracheally intubated patients at 9 hospitals was 11.5% by patient and 6.6% by mobilization session. These occurrence rates were higher than those found in previous studies that included non-mechanically ventilated patients and patients with in-bed exercise. The highest activity level was identified as a risk factor for PSE occurrence, and close vigilance is required during mobilization in the standing position with regard to circulatory dynamics.

## Figures and Tables

**Table 1:** Baseline characteristics and outcomes of patients undergoing mobilization sessions.

	All Patients (*n* = 87)	Group without Patient-Related Safety Events (*n* = 77)	Group with Patient-Related Safety Events (*n* = 10)	*p*-Value
Baseline characteristics				
Age in years; median (IQR)	72.0 (61.0–79.0)	69.0 (60.0–79.0)	76.0 (70.0–80.0)	0.401
Sex: male; *n* (%)	55 (63%)	49 (64%)	6 (60%)	1
BMI; kg/m^2^; median (IQR)	22.8 (19.8–26.1)	23.1 (20.0–27.0)	20.7(19.5–23.0)	0.084
Charlson Comorbidity Index; median (IQR)	2 (1–2)	2 (1–2)	2 (0–2)	0.508
Barthel Index prior to hospital admission; median (IQR)	100.0 (90.0-100.0)	100.0 (95.0-100.0)	100.0 (25.0-100.0)	0.325
ICU admission diagnosis				0.837
Sepsis; *n*	23	21	2	
Gastrointestinal condition; *n*	21	17	4	
Cardiac failure including post cardiosurgery; *n*	16	15	1	
Respiratory failure; *n*	13	11	2	
Trauma; *n*	5	5	0	
Other; *n*	9	8	1	
APACHE II score; median (IQR)	27.0 (21.0–30.0)	25.0 (21.0–30.0)	27.0 (23.0–29.0)	0.995
SOFA at ICU admission; median (IQR)	10.0 (8.0–14.0)	10.0 (8.0–14.0)	9.00 (8.0–10.0)	0.161
Outcomes				
Time to first mobility session in days; median (IQR)	2.0 (1.0–3.0)	2.0 (1.0–4.0)	1.0 (0.0–1.0)	0.002 *
Time to first getting out of bed in days; median (IQR)	4.0 (3.0–6.0)	4.0 (3.0–7.0)	2.5 (2.0–4.5)	0.041 *
Time from first mobility session to first getting out of bed in days; median (IQR)	1.0 (1.0–3.0)	1.0 (0.0–3.0)	1.5 (1.0–5.0)	0.397
Length of mechanical ventilation in days; median (IQR)	6.0 (4.0–10.0)	6.0 (4.0–10.0)	5.7 (4.0–11.3)	0.882
Length of ICU stay in days; median (IQR)	10.0 (7.0–14.0)	10.0 (6.0–14.0)	14.0 (7.0–17.0)	0.219
Length of hospital stay in days; median (IQR)	42.0 (26.0–71.0)	41.0 (25.5–70.5)	53.5 (26.8–119.3)	0.354
Barthel Index at the time of hospital discharge; median (IQR)	70.0 (10.0–100.0)	65.0 (5.0–100.0)	75.0 (65.0–95.0)	0.262
Reintubation during hospital stay; *n* (%)	15 (17%)	14 (18%)	1 (10%)	1
ICU re-admission; *n* (%)	10 (11%)	10 (13%)	0 (0%)	0.638

IQR: interquartile range; BMI: body mass index; ICU: Intensive Care Unit; APACHE: Acute Physiology and Chronic Health Evaluation; SOFA: Sequential Organ Failure Assessment. * *p* < 0.05.

**Table 2:** Comparison of mobilization sessions with and without patient-related safety events.

Parameters	Mobility Sessions without Patient-Related Safety Event(s) (*n* = 185)	Mobility Sessions with Patient-Related Safety Event(s) (*n* = 13)	*p*-Value
Mode of mechanical ventilation			0.475
Assist and Control; *n*	29	4	
SIMV; *n*	37	1	
PEEP and PS; *n*	115	8	
Other; *n*	4	0	
Parameters of mechanical ventilation			
F_I_O_2_ (10^2^); median (IQR)	35 (30–40)	35 (35–40)	0.495
PEEP; median (IQR)	6 (5–8)	8 (5–8)	0.529
Treatment			
Patients on vasopressors before active mobilization; *n* (%)	33 (18%)	6 (46%)	0.024 *
Undergoing CRRT; *n* (%)	13 (7%)	0 (0%)	0.606
RASS; median (IQR)	−1 (–1–0)	0 (0–0)	0.045 *
Highest activity level	3 (3–3)	4 (4–4)	<0.001 *
Vital signs before mobility session			
PaO_2_ (mmHg); median (IQR)	94 (80–118)	102 (93–120)	0.473
PaCO_2_ (mmHg); median (IQR)	39 (36–44)	39 (37–46)	0.134
SBP (mmHg); median (IQR)	125 (111–140)	111 (101–129)	0.144
Heart rate (beats/min); median (IQR)	90 (80–100)	90 (80–97)	0.946
Respiratory rate (breaths/min); median (IQR)	18 (15–21)	17 (14–20)	0.68
Body temperature (°C); median (IQR)	37.3 (36.8–37.9)	37.7 (36.8–38.4)	0.26
Profession of mobilization provider			
Doctor; *n* (%)	52 (28%)	3 (23%)	1
Nurse; *n* (%)	153 (83%)	13 (100%)	1
Physical therapist; *n* (%)	175 (95%)	10 (77%)	0.043 *
Occupational therapist; *n* (%)	28 (15%)	3 (23%)	0.433

SIMV: synchronized intermittent mandatory ventilation; PS: pressure support; PEEP: positive end-expiratory pressure; IQR: interquartile range; RASS: Richmond Agitation–Sedation Scale; CRRT: continuous renal replacement therapy; SBP: systolic blood pressure. Highest activity level is shown based on mobility protocol. * *p* < 0.05.

**Table 3:** Types and timing of patient-related safety events.

	Number of Patient-Related Safety Events; *n*	Timing of Patient-Related Safety Event Occurrence in Days; Median (IQR)	Event Rate per 1000 Sessions
Type of patient-related safety event			
Systolic blood pressure	8	5 (3–7)	40.4
Heart rate	3	2 (2–3)	15.2
Desaturation	2	5.5 (5–6)	10.1
Timing of patient-related safety event			
Level 3: Sitting on edge of bed	4	4 (3–4)	26.1
Level 4: Standing	8	5 (2–6)	205.1
Level 5: Walking	1	7 (7–7)	166.7

A total of 198 mobilization sessions were carried out. Ten patients experienced 13 patient-related safety events. Eight patients experienced one patient-related safety event once. One patient experienced a patient-related safety event twice, and both events were associated with systolic blood pressure. One patient experienced a patient-related safety event three times, and all of these events were associated with systolic blood pressure.

**Table 4:** Risk factors for the occurrence of patient-related safety events.

	Odds Ratio	*p*-Value
Use of vasopressors	2.46 (0.70–8.63)	0.159
RASS	1.34 (0.63–2.86)	0.448
Highest activity level	2.83 (1.13–7.04)	0.026 *
Mobility session without Physical Therapist	0.37 (0.08–1.79)	0.218

RASS: Richmond Agitation–Sedation Scale * *p* < 0.05.

## Data Availability

The data presented in this research are available upon request from the corresponding author after discussion with the Institutional Review Board of the Japanese Red Cross Nagaoka Hospital.

## Supplementary Materials

The following supplementary materials are available online at https://www.mdpi.com/article/10.3390/jcm10122607/s1: Table S1. Hospital Background Information; Table S2. Mobilization Protocol; Table S3. The Study Management Committee.
