# Supplementary material for: Risk Factors of Patient-Related Safety Events during Active Mobilization for Intubated Patients in Intensive Care Units—A Multi-Center Retrospective Observational Study
Source: J Clin Med. 2021 Jun 13;10(12):2607. doi: 10.3390/jcm10122607 (PMC8231849; doi:10.3390/jcm10122607)
Supplement: Supplementary file 1 [file jcm-10-02607-s001.zip › jcm-1234625-proofed-supplementary.pdf]

Table S1. Hospital background information.

Facility Name	A	B	C	D	E	F	G	H	I
Number of beds	868	740	740	592	500	464	464	415	376
Number of ICU beds	20	26	8	12	6	24	8	8	10
ICU system	Closed	Mandatory	Mandatory	Closed	Closed	Open	Elective	Open	Mandatory
Patient-to-nurse ratio	2:1	2:1	2:1	1:1	2:1	2:1	2:1	2:1	2:1
Dedicated physiotherapist	Yes	Yes	Yes	No	No	No	Yes	Yes	No
Mobilization protocol	Yes	Yes	Yes	Yes	Yes	Yes	Yes	Yes	Yes
Sedation protocol	No	Yes	No	No	No	No	Yes	No	No
Agitation protocol	No	Yes	No	No	No	No	No	No	No
SAT/SBT protocol	Yes	Yes	Yes	No	No	No	Yes	No	Yes

Closed: intensivist decides therapeutic policy; Mandatory: intensivist is involved in deciding treatment policy; Elective: intensivist is mainly involved as a consultant; Open: physicians in each department decide therapeutic policy [1].

Table S2. Mobilization protocol [2].

Level 1

Respiratory	Criteria for proceeding to the next step
☐ Turning	☐ Oxygenation/hemodynamic stability
☐ RHOB ≤ 45 degrees	☐ Can withstand posture change
☐ Passive ROM exercise	☐ Can withstand RHOB ≤ 45 degrees
☐ Respiratory physical therapy	

Level 2

Raise Head of Bed	Criteria for proceeding to the next step
☐ Turning	☐ All Level 1 exercises can be carried out
☐ RHOB ≤ 60 degrees	☐ Can withstand RHOB ≤ 60 degrees
☐ Passive ROM exercise	☐ Anti-gravity movement possible
☐ Active ROM exercise	
☐ Respiratory physical therapy	
☐ Bed mobility exercise	

Level 3

Sitting	Criteria for proceeding to the next step
☐ Turning	☐ All Level 2 exercises can be carried out
☐ RHOB ≤ 60 degrees	☐ Can withstand sitting on the edge of the bed
☐ Passive ROM exercise	
☐ Active ROM exercise	
☐ Bed mobility exercise	
☐ Sitting on the edge of the bed	

Level 4

Standing	Criteria for proceeding to the next step
☐ Turning	☐ All Level 3 exercises can be carried out
☐ RHOB ≤ 60 degrees	☐ Can withstand partial weight standing
☐ Passive ROM exercise	
☐ Active ROM exercise	
☐ Standing at the side of the bed	
☐ Stand and transfer to a chair	

Level 5

Walking	Criteria for proceeding to the next step
☐ Turning	☐ Increase walking distance gradually
☐ RHOB ≤ 60 degrees	
☐ Passive ROM exercise	
☐ Active ROM exercise	
☐ Walking with assistance	
☐ Walking without assistance	

Table S3. The study management committee.

The Study Management Committee

◆ Fukuyama City Hospital

· Yoshiyuki Teranobu
· Shuhei Ikeguchi

◆ Hiroshima University Hospital

· Shunsuke Taito

◆ Japanese Red Cross Ishinomaki Hospital

· Shuntaro Suzuki

◆ Japanese Red Cross Maebashi Hospital

· Kenji Fujizuka
· Takeshi Mizuno
· Kei Tsunoda
· Emi Abe

◆ Japanese Red Cross Nagaoka Hospital

· Tomoaki Terasawa

◆ Japanese Society for Early Mobilization

· Sho Iida
· Tomoya Kuroda

◆ Nagasaki University

· Tsubasa Watanabe

◆ Naha City Hospital

· Takuya Tonaki
· Shinya Kawabata
· Yoshifumi Heshiki
· Yousuke Satoi
· Hiroshi Tomiyama
· Yuji Miyata

◆ National Hospital Organization Nagoya Medical Center

· Mika Ohno
· Yayoi Honjyo

◆ Showa University School of Medicine

· Fumihito Kasai
· Maiko Mori
· Hiroko Kato
· Kota Kubodera
· Kaori Tsuruta

◆ Tokyo Women's Medical University

· Tetsuo Ikai
· Tatsuya Horibe
· Gen Kudo
· Masako Shimada
· Syouhei Yokota
· Naoko Shima

◆ Yao Tokushukai General Hospital

· Hiromasa Harada
· Yasuko Muranaka

◆ Yuuai Medical Center

· Masahiro Tamashiro
· Shohei Miyazato
· Shogo Sakihama
· Ryo Nagato

Reference

1. Pronovost, P.J.; Angus, D.C.; Dorman, T.; Robinson, K.A.; Dremsizov, T.T.; Young, T.L. Physician staffing patterns and clinical outcomes in critically ill patients: A systematic review. *JAMA* **2002**, *288*, 2151–2162.
2. Watanabe, S.; Kotani, T.; Taito, S.; Ota, K.; Ishii, K.; Ono, M.; Katsukawa, H.; Kozu, R.; Morita, Y.; Arakawa, R.; et al. Determinants of gait independence after mechanical ventilation in the intensive care unit: A Japanese multicenter retrospective exploratory cohort study. *J. Intensive Care* **2019**, *7*, 53.
